# Berbamine inhibits RANKL- and M-CSF-mediated osteoclastogenesis and alleviates ovariectomy-induced bone loss

**DOI:** 10.3389/fphar.2022.1032866

**Published:** 2022-11-02

**Authors:** Guobin Qi, Zengxin Jiang, Wei Lu, Defang Li, Weibing Chen, Xiuying Yang, Lei Ding, Hengfeng Yuan

**Affiliations:** ^1^ Department of Orthopaedics, Shanghai Sixth People’s Hospital, Shanghai, China; ^2^ Department of Orthopedic Surgery, Zhongshan Hospital, Fudan University, Shanghai, China; ^3^ Department of Orthopedic Surgery, Shanghai TCM-Integrated Hospital Shanghai University of TCM, Shanghai, China; ^4^ Department of Orthopedic Surgery, Jinshan Hospital, Fudan University, Shanghai, China; ^5^ Department of Radiology, Jinshan Hospital, Fudan University, Shanghai, China

**Keywords:** berbamine, osteoclastogenesis, osteoporosis, NF-κB, MAPK

## Abstract

Osteoporosis is a common public health problem characterized by decreased bone mass, increased bone brittleness and damage to the bone microstructure. Excessive bone resorption by osteoclasts is the main target of the currently used drugs or treatment for osteoporosis. Effective antiresorptive drugs without side effects following long-term administration have become a major focus of anti-osteoporotic drugs. In the present study, we investigated the effect of berbamine, a small molecule natural product from *Berberis amurensis Rupr*, a traditional Chinese medicine, on RANKL-induced osteoclast differentiation *in vitro* and ovariectomy-induced bone loss *in vivo*. The results demonstrated that berbamine at a safe and effective dose inhibited osteoclastogenesis and bone resorption function *in vitro* by suppressing the nuclear factor-κB signaling pathway. In addition, berbamine protected against osteoporosis by inhibiting osteoclastogenesis and bone resorption function without affecting osteogenesis in the ovariectomy mouse model. These findings revealed that berbamine has a protective role against osteoporosis and may represent a novel promising treatment strategy for osteoporosis.

## Introduction

With the rapid growth of aging populations, osteoporosis has become a common public health problem, threatening more than 200 million people all over the world (Reginster and Burlet, 2006). Osteoporosis is a systemic bone disease characterized by decreased bone mass, increased bone brittleness and damage to the bone microstructure, which result in an increased risk of fractures (Seeman, 2002; Li et al., 2019). The development of osteoporosis is mainly attributed to excessive bone resorption by osteoclasts (Teitelbaum, 2000; Zaidi, 2007), which has become the main target of the currently used drugs or treatment for osteoporosis, such as bisphosphonates and denosumab. However, most of these antiresorptive drugs have limitations and side effects, including atypical femur fracture, multiple vertebral fractures and medication-related osteonecrosis of the jaw (Borggaard et al., 2022; Jung et al., 2022; Kendler et al., 2022), which affect their long-term administration and patient adherence. Therefore, effective bone resorption inhibitors without side effects following long-term administration have become a major focus for the treatment of osteoporosis.

Osteoclasts, tartrate-resistant acid phosphatase (TRAP)-positive multinuclear cells, are derived from the monocyte/macrophage lineage and play an important role in bone resorption and continuous bone remodeling (Boyle et al., 2003). The receptor activator of nuclear factor-κB (RANK) ligand (RANKL) and macrophage colony-stimulating factor (M-CSF) are both necessary and sufficient for osteoclast differentiation from monocytic precursor cells (Boyle et al., 2003; Asagiri and Takayanagi, 2007). Activation of RANK on the cell membrane of preosteoclasts by RANKL drives osteoclast differentiation by activating the tumor necrosis factor receptor-associated factor 6 (TRAF6)-mediated nuclear factor-κB (NF-κB), mitogen-activated protein kinase (MAPK) and calcium signaling pathways (Nakashima et al., 2011). Nuclear factor of activated T cells 1 (NFATc1), a major regulator of osteoclastogenesis, is subsequently activated and induces the expression of osteoclast-specific genes, such as those encoding cathepsin K (CTSK), TRAP, and matrix metalloproteinase 9 (MMP9) (Gohda et al., 2005; Takayanagi, 2007). Therefore, RANKL-mediated signaling pathways are considered promising therapeutic targets of osteoclast-associated diseases, in which excessive generation of osteoclasts or increased osteoclast activity occurs.

Berbamine (BBM), a nonbasic quaternary benzylisoquinoline plant alkaloid from the traditional Chinese medicine, *Berberis amurensis Rupr*, is used clinically in patients with a low leukocyte count caused by chemotherapy or radiotherapy (Salehi et al., 2019). In addition, BBM has been reported to have several pharmacological activities, such as antiarrhythmic, antitumor, antipyretic and anti-inflammatory effects (Ren et al., 2008; Parekh et al., 2009; Hu et al., 2020). Increasing evidence shows that BBM negatively regulates the NF-κB signaling pathway (Liang et al., 2009; Liu et al., 2020; Han et al., 2021), which plays a critical role during the process of osteoclastogenesis. Therefore, we hypothesized that BBM may be beneficial for the treatment of osteoporosis by downregulating osteoclastogenesis and osteoclast activity through NF-κB signaling pathway. In the present study, we investigated the effect of BBM on RANKL-induced osteoclast differentiation from bone marrow-derived macrophages (BMMs) *in vitro* and ovariectomy (OVX)-induced bone loss *in vivo*. The results demonstrated that BBM inhibits osteoclastogenesis and osteoclast activity by suppressing the NF-κB signaling pathway, thereby preventing bone loss in an OVX-induced osteoporosis mouse model.

## Materials and methods

### Reagents and antibodies

BBM was purchased from Selleck (Shanghai, China) and dissolved in DMSO (Sigma–Aldrich, St. Louis, MO, United States). Fetal bovine serum (FBS), penicillin–streptomycin (P/S) and minimum essential medium α (α-MEM) were purchased from Gibco (Grand Island, NY, United States). M-CSF and RANKL were purchased from R&D Systems (Minneapolis, MN, United States). Primary antibodies against c-Fos (CST, #2250, diluted at 1:1,000), NFATc1 (CST, #8032, diluted at 1:1,000), p-JNK (Thr183/Tyr185, CST, #4668, diluted at 1:1,000), JNK (CST, #9252, diluted at 1:1,000), p-ERK (Thr202/Tyr204, CST, #4370, diluted at 1:1,000), ERK (CST, #4695, diluted at 1:1,000), p-P38 (Thr180/Tyr182, CST, #4511, diluted at 1:1,000), P38 (CST, #8690, diluted at 1:1,000), p-IκBα (Ser32, CST, #2859, diluted at 1:1,000), IκBα (CST, #4812, diluted at 1:1,000), p-P65 (Ser536, CST, #3033, diluted at 1:1,000), P65 (CST, #8242, diluted at 1:1,000) and β-actin (CST, #4970, diluted at 1:1,000) were purchased from Cell Signaling Technology (Danvers, MA, United States). Antibodies against CTSK (Abcam, ab19027, diluted at 1:1,000) and MMP9 (Abcam, ab38898, diluted at 1:1,000) were purchased from Abcam (Cambridge, MA, United States). The secondary antibodies against mouse or rabbit (diluted at 1:1,000) were purchased from Beyotime (Shanghai, China).

### Cell culture

BMMs were isolated from the femurs and tibias of C57BL/6J mice aged 6–8 weeks as previously described (Tang et al., 2021). Briefly, we removed the femurs and tibias from euthanized mice under aseptic conditions. Bone marrow cells in the bone marrow cavity were flushed out and maintained in α-MEM supplemented with 10% (v/v) FBS and 1% P/S. After 16 h, supernatant cells were collected and cultured in medium supplemented with 50 ng/mL M-CSF. Adherent cells were harvested and classified as BMMs. In all experiments, BMMs were maintained in the presence of 50 ng/mL M-CSF in an incubator at 37°C and 5% CO_2_. In selected experiments, 50 ng/ml RANKL was added to stimulate osteoclast differentiation.

The MC3T3-E1 murine preosteoblast cell line was purchased from the Cell Bank of Chinese Academy of Sciences (Shanghai, China) and maintained in ɑ-MEM supplemented with 10% FBS and 1% P/S in an incubator at 37°C and 5% CO_2_.

### Cytotoxicity assay

To examine the toxicity of BBM at different concentrations on BMMs and MC3T3-E1 cells, a Cell Counting Kit-8 (CCK-8) test was performed according to the manufacturer’s instructions. In brief, BMMs and MC3T3-E1 cells were cultured in 96-well plates (5×10^3^ cells per well) for 24 h and then treated with various concentrations of BBM for 2, 4 or 6 days. At each time point, 10 μl of CCK8 reagent was added to each well and incubated at 37 °C. After 2 h, the optical density value at 450 nm (OD450) was measured using a microplate reader (BioTek, Winooski, VT, United States).

### *In vitro* osteoclastogenesis assay

BMMs were cultured in 96-well plates (5×10^3^ cells per well) for 24 h and treated with 50 ng/ml RANKL for another 5–7 days with or without BBM at varying concentrations (0.5, 1, 2, and 4 μM). To determine the time-dependent effect of BBM on osteoclastogenesis, BMMs were exposed to BBM at the early stage (D1-2, Day 1 and Day 2), middle stage (D3–4, Day 3 and Day 4), late stage (D5–6, Day 5 and Day 6) and all stages (D1–6, Day 1 to Day 6). The medium was changed every 2 days until mature osteoclasts formed, which were classified as TRAP-positive (TRAP^+^) and multinucleated cells. TRAP staining assays were conducted using a leukocyte acid phosphatase kit (Sigma–Aldrich) according to the manufacturer’s protocol. TRAP^+^ cells with ≥3 nuclei were identified as mature osteoclasts, and the number was counted under an inverted microscope (Olympus, Japan). TRAP enzyme activity was determined by means of a TRAP Assay Kit (Beyotime). Cell culture supernatants were collected and incubated with working solution for 10 min at 37°C according to the manufacturer’s instructions. The absorbance at 405 nm (A_405_) was measured using a microplate reader (BioTek).

### F-actin ring staining

To assess the effect of BBM on the cytoskeletal structure of osteoclasts, BMMs were cultured on bovine bone slides and induced by adding BBM (0, 2 and 4 μM) and 50 ng/ml RANKL for 5–7 days. For F-actin ring staining, the preosteoclasts or mature osteoclasts on bone slides were fixed and stained with Alexa Fluor 488-labeled phalloidin for 1 h followed by DAPI staining to visualize cellular nuclei. Fluorescence imaging was performed using a confocal laser-scanning microscope (Olympus FV3000, Japan).

### Resorption pit formation assay

The pit formation assay was used to measure osteoclast bone resorption activity. BMMs were cultured in collagen-coated plates (Corning, NY, United States) and treated with 50 ng/mL M-CSF and 50 ng/ml RANKL until mature osteoclasts were formed. The osteoclasts were collected by digestion using cell dissociation solution (Sigma–Aldrich) and seeded on bovine bone slides followed by 48 h of incubation with or without BBM. After osteoclasts were gently brushed away from the bone slides with a brush, a scanning electron microscope (SEM; SU8100, Hitachi, Japan) was used to visualize the resorption pits, and the relative resorption area was quantified using ImageJ software.

### Nuclear translocation of NFATc1 and P65

BMMs were cultured in confocal culture dishes (1×10^5^ cells per well) for 24 h to allow adherence to the plate and treated with or without 4 μM BBM for 2 h followed by incubation with 50 ng/ml RANKL for 30 min. BMMs were then fixed with 4% PFA for 10 min, permeabilized with 0.1% Triton X-100 (Sigma–Aldrich) for 5 min and blocked with 4% BSA (Sigma–Aldrich) for 1 h. Finally, cells were incubated with NFATc1 or P65 antibody at 4°C overnight followed by incubation with Alexa Fluor 488-labeled secondary antibody at room temperature for 1 h. DAPI staining (ThermoFisher Scientific, MA, United States) was used to visualize cellular nuclei, and fluorescence imaging was performed using a confocal microscope (Olympus).

### *In vitro* osteoblast differentiation assay

MC3T3-E1 cells were cultured in osteogenic differentiation medium containing 100 nM dexamethasone, 50 μg/ml ascorbic acid and 10 mM β-glycerophosphate. The medium was changed every 2 days, and osteoblast differentiation was assessed by alkaline phosphatase (ALP) staining (Solarbio, Beijing, China) and Alizarin Red S (ARS) staining (Solarbio) according to the manufacturer’s instructions on Day 7 and Day 21, respectively. Images were acquired using an inverted microscope (Olympus).

### Western blotting

To examine the expression of osteoclast-specific proteins, BMMs were induced with 50 ng/ml RANKL with or without BBM for the indicated time. To examine signaling pathways involved in osteoclastogenesis, BMMs were pretreated with 4 μM BBM for 1 h and induced with 50 ng/ml RANKL for the indicated time. Cells were lysed on ice for 15 min with RIPA buffer (Beyotime), and protein concentration was measured using an BCA Protein Assay Kit (Beyotime). Approximately 20 μg of protein lysate was subjected to sodium dodecyl sulfate–polyacrylamide gel electrophoresis (SDS–PAGE) on 10% gels to separate proteins followed by transfer to polyvinylidene difluoride (PVDF) membranes (Millipore, Bedford, MA, United States). The membranes were blocked with 5% skimmed milk for 1 h and incubated with a specific primary antibody overnight at 4°C. The membranes were then incubated with HRP-conjugated secondary antibodies for 1 h. Images were visualized using a chemiluminescent imaging system (Tanon, Shanghai, China) and enhanced chemiluminescence (ECL) (Epizyme, Shanghai, China). The relative intensities of the protein bands were quantified using ImageJ software.

### Quantitative real-time PCR

BMMs were treated with different concentrations of BBM in the presence of 50 ng/ml RANKL for 5 days, and the mRNA levels of osteoclast-specific genes were detected by quantitative real-time PCR. Total RNA samples were isolated using TRIzol reagent (Invitrogen, Carlsbad, CA, United States) and reverse-transcribed into cDNA using PrimeScript RT Master Mix (Takara, Tokyo, Japan) according to the manufacturer’s protocol. Quantitative real-time PCR was performed using SYBR Premix ExTaq (Takara) on a QuantStudio 5 Real-Time PCR System (Applied Biosystems, Foster City, CA, United States). Fold change was calculated using 2^−ΔΔCT^. The GAPDH housekeeping gene was used as an internal control. The sequences of the primers used in this study are listed in Table 1.

**TABLE 1:** Primer sequences used for quantitative real-time PCR.

Gene	Forward (5′-3′)	Reverse (5′-3′)
NFATc1	CCC​GTC​ACA​TTC​TGG​TCC​AT	CAA​GTA​ACC​GTG​TAG​CTG​CAC​AA
CTSK	ATG​TGG​GTG​TTC​AAG​TTT​CTG​C	CCA​CAA​GAT​TCT​GGG​GAC​TC
TRAP	CGC​TTC​AAA​ATT​CCA​CGT​ACA​A	CAG​CAT​CAC​TGT​GTC​CAG​CAT
MMP9	GCT​GAC​TAC​GAT​AAG​GAC​GGC​A	TAG​TGG​TGC​AGG​CAG​AGT​AGG​A
GAPDH	ACC​CAG​AAG​ACT​GTG​GAT​GG	TTC​AGC​TCA​GGG​ATG​ACC​TT

### OVX mouse model

All mouse experimental procedures were approved by the Ethics Committee of Jinshan Hospital, Fudan University. Female C57BL/6J mice (6–8 weeks of age) were supplied by Shanghai Slac Laboratory Animal Co., Ltd. (Shanghai, China) and maintained in a specific pathogen-free animal facility. After 1 week of acclimatization, mice were randomly assigned to the following treatment groups: sham group, OVX group, OVX + low-dose (25 mg/kg) BBM group and OVX + high-dose (50 mg/kg) BBM group. For the OVX-induced bone loss model, mice were anesthetized and subjected to bilateral ovariectomy or sham ovariectomy followed by intraperitoneal injection of DMSO or BBM once every 2 days for 8 weeks. The bone mineral density (BMD) measurements of the lumbar spine, femur and total body were determined by full-body scans of mice using dual-energy X-ray absorptiometry (DXA; OsteoSys, Seoul, Korea). The head was excluded from the analysis of total body BMD.

### Microcomputed tomography analysis

After the mice were euthanized by cervical dislocation, femurs were harvested and fixed in 4% PFA for 48 h. Subsequent µCT analysis was conducted to evaluate parameters of the trabecular bone in the distal metaphysis of the femurs using a Scanco µCT-40 scanner (Scanco Medical, Bassersdorf, Switzerland). Images were acquired at 55 kV and 145 mA with an isotropic voxel size of 6 μm, and 300 slices (1.8 mm) proximal to the growth plate were selected as the regions of interest (ROIs) for analysis. Bone parameters, including bone volume fraction (BV/TV), trabecular number (Tb.N), trabecular thickness (Tb.Th), and trabecular separation (Tb.Sp) were analyzed using NRecon software (version 1.6, Bruker), CTAn software (version 1.9, Bruker) and CTVol software (version 2.0, Bruker).

### Histopathological and immunohistochemical analysis

Femurs were decalcified using 10% EDTA solution for 10 days and then embedded in paraffin. Sections were cut (approximately 5 μm) and stained with hematoxylin and eosin (H&E), Masson’s trichrome or TRAP staining. Tissue samples from the heart, liver, spleen, lungs and kidneys were also collected and stained with H&E. For IHC analysis, sections were incubated with anti-OCN (Abcam) antibodies overnight at 4°C followed by incubation with secondary biotinylated antibody for 1 h at room temperature. The immunoreaction was visualized with a 3,3′-diaminobenzidine (DAB) Substrate Kit (Abcam), and nuclei were counterstained with hematoxylin. Images were acquired using an inverted microscope (Olympus). The number of osteoclasts per bone surface (N.Oc/BS) and osteoclast surface per bone surface (Oc.S/BS) were determined in TRAP-stained sections using ImageJ software.

### Dynamic bone histomorphometry

Calcein–Alizarin red S labeling was performed to measure dynamic bone formation. Briefly, mice were intraperitoneally injected with 20 mg/kg calcein (Sigma–Aldrich) and 20 mg/kg Alizarin red S (Sigma–Aldrich) at 10 and 2 days before euthanasia. Undecalcified bones were embedded in methyl methacrylate (Sigma–Aldrich) and cut into 10-μm thick sections. The double labeling was then observed using a fluorescence microscope (Leica, Nussloch, Germany). The mineral apposition rate (MAR), mineralizing surface/bone surface (MS/BS) and bone formation rate/bone surface (BFR/BS) were determined using ImageJ software.

### Enzyme-linked immunosorbent assay

After the mice were euthanized by cervical dislocation, blood samples were collected by cardiac puncture and centrifuged at 3,500 ×g for 15 min at 4°C to obtain serum. The serum concentrations of osteocalcin (OCN) and C-terminal telopeptide of type I collagen (CTX-1) were quantified using a commercial ELISA kit (Nanjing Jiancheng Bioengineering Institute, Nanjing, China). All procedures were performed according to the manufacturer’s instructions.

### Statistical analysis

Data are shown as the mean ± standard deviation (SD). The unpaired two-tailed Student’s t test was used for experiments comparing two groups, and one-way analysis of variance (ANOVA) was used to compare more than two groups. For all experiments, *p* < 0.05 was considered statistically significant and represented by “*”, and *p* < 0.01 was represented by “**”. Statistical analysis was performed using GraphPad Prism software, version 9 (GraphPad Software Inc.).

## Results

### BBM suppresses RANKL-Induced osteoclastogenesis *in vitro*

**FIGURE 1:**
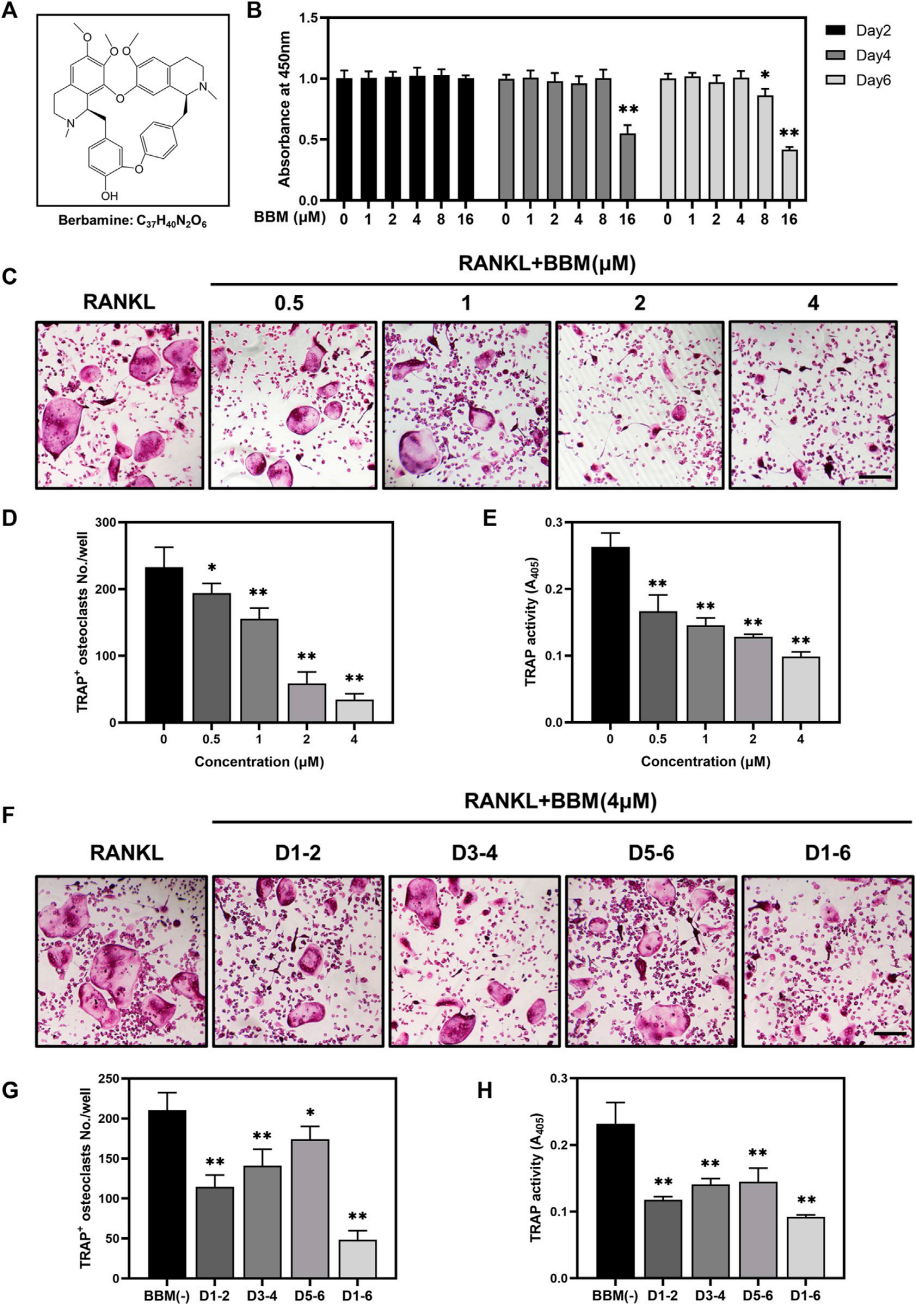
BBM suppresses RANKL-induced osteoclastogenesis *in vitro*. **(A)** Chemical structure of BBM. **(B)** BMMs were treated with BBM (0, 1, 2, 4, 8, and 16 μM) for 2, 4, and 6 days. Cell viability was determined by CCK-8 assay. **(C)** Representative images of TRAP staining in BMMs cultured with RANKL (50 ng/ml) and the indicated concentrations of BBM for 5–7 days (scale bar = 200 μm). **(D,E)** Quantitative analysis of the number of mature osteoclasts **(D)** and TRAP activity in the culture supernatant **(E)**. **(F)** Representative images of TRAP staining in BMMs cultured with RANKL (50 ng/ml) and 4 μM BBM for the indicated period (scale bar = 200 μm). **(G,H)** Quantitative analysis of the number of mature osteoclasts **(G)** and TRAP activity in the culture supernatant **(H)**. *p < 0.05 and **p < 0.01 compared to the control group.

To assess the toxic effects of BBM at varying concentrations (Figure 1A) on the viability of BMMs, CCK-8 assays were performed, and no obvious cytotoxicity was observed at BBM concentrations ranging from 1 to 4 μM after 6 days of incubation (Figure 1B). Therefore, the highest concentration of BBM applied in subsequent experiments was 4 μM. To identify the effect of BBM on osteoclastogenesis *in vitro*, BMMs were cultured with 50 ng/ml RANKL with or without BBM (0.5, 1, 2 and 4 μM) for 5–7 days. BBM significantly decreased the number of mature osteoclasts and TRAP activity in the culture supernatant in a concentration-dependent manner with the lowest concentration (0.5 μM) exhibiting an inhibitory effect (Figures 1C–E). Furthermore, BMMs were exposed to BBM at different stages of osteoclast differentiation to assess the time-dependent effects. TRAP staining and TRAP activity assays showed that BBM exhibited the strongest inhibitory effect on osteoclastogenesis at the early stage (Figures 1F–H). Collectively, these data demonstrated that BBM suppresses RANKL-induced osteoclastogenesis in a concentration- and time-dependent manner *in vitro*.

### BBM inhibits RANKL-Induced F-Actin ring formation and osteoclast resorption function *in vitro*

**FIGURE 2:**
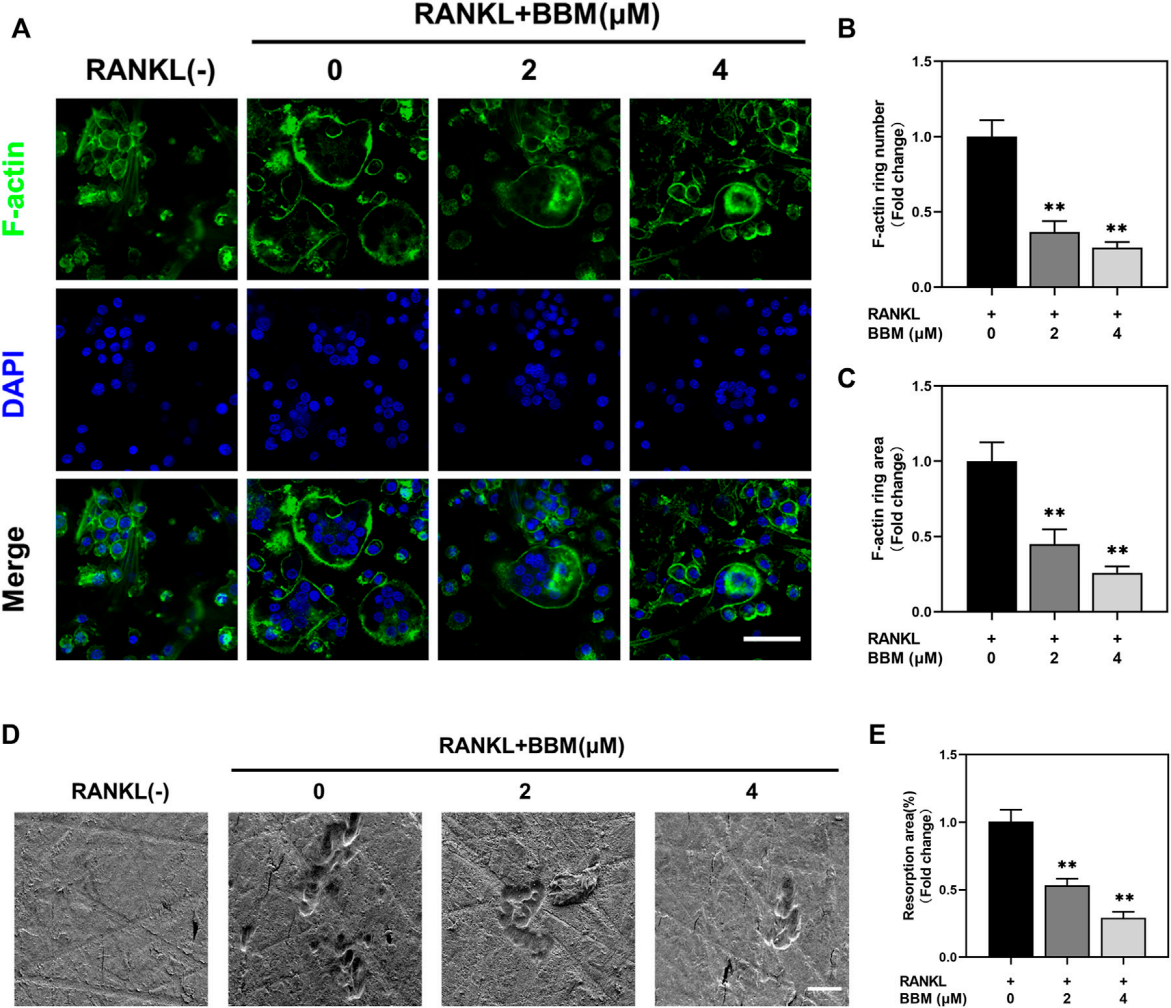
BBM inhibits RANKL-induced F-actin ring formation and osteoclast resorption function *in vitro*. **(A)** Representative confocal images of F-actin ring staining of BMMs cultured on bone slides with or without RANKL (50 ng/ml) and BBM (0, 2, and 4 μM) for 5–7 days (scale bar = 50 μm). **(B–C)** Quantitative analysis of the number **(B)** and area **(C)** of F-actin rings. **(D)** Representative SEM images of resorption pits on bone slides (scale bar = 50 μm). **(E)** Quantitative analysis of the resorption area. *p < 0.05 and **p < 0.01 compared to the control group.

To assess the effect of BBM on the formation of F-actin rings, which are essential for bone resorption, BMMs were induced to differentiate into osteoclasts on bovine bone slides and stained with fluorescently labeled phalloidin (Figure 2A). Osteoclasts with intact F-actin rings were formed after RANKL stimulation, while BBM at a concentration of 2 or 4 μM significantly decreased the F-actin ring number and F-actin ring area (Figures 2B,C).

To further assess whether BBM affects the bone resorption function of mature osteoclasts, resorption pit formation assays were performed by seeding equal numbers of osteoclasts on bovine bone slides. Quantitative analysis of the bone resorption area showed that the bone resorption activity of mature osteoclasts was significantly reduced by BBM (Figures 2D,E).

### BBM suppresses osteoclast-specific gene expression

**FIGURE 3:**
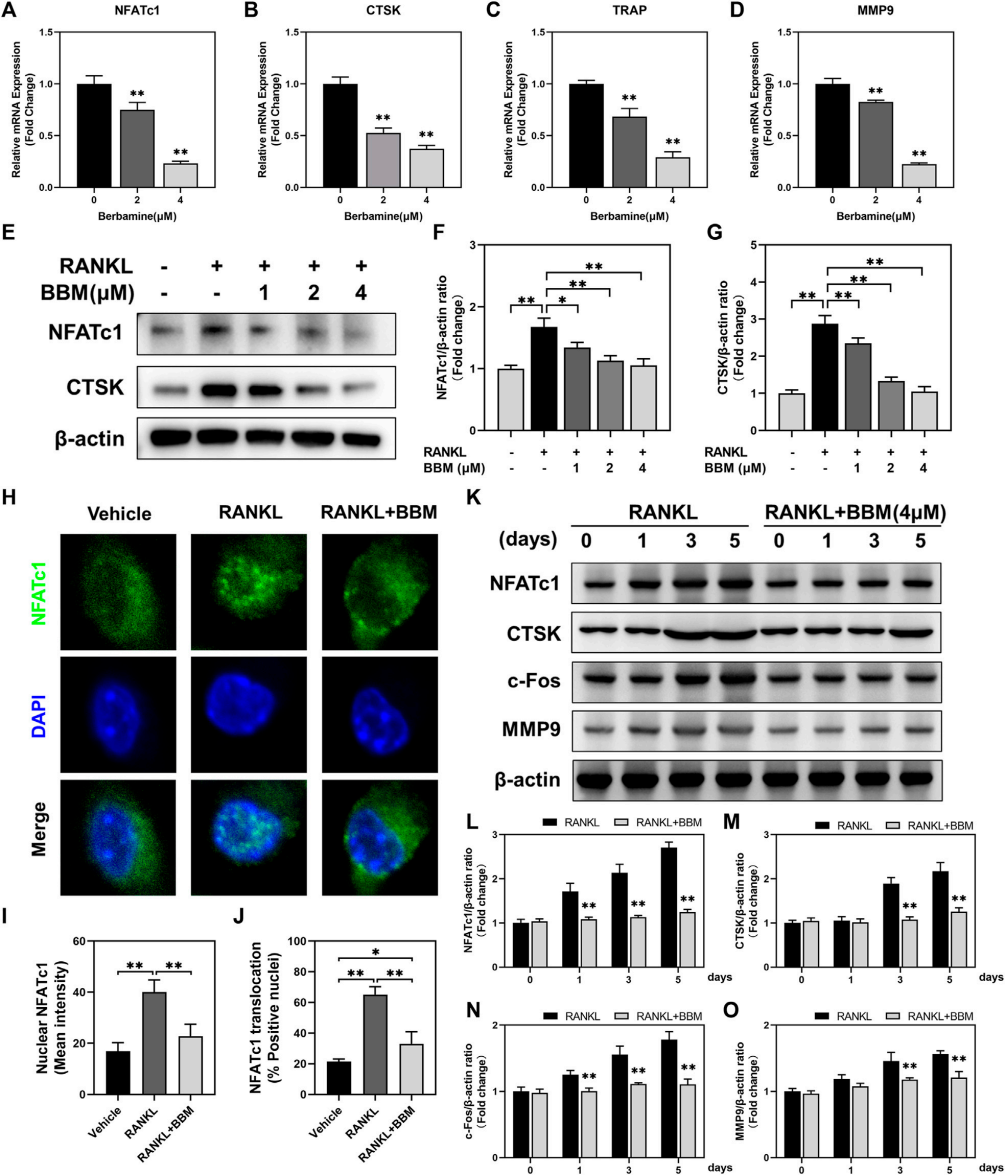
BBM suppresses the expression of osteoclast-specific genes. **(A–D)** The mRNA expression levels of NFATc1 **(A)**, CTSK **(B)**, TRAP **(C)** and MMP9 **(D)** were determined by quantitative real-time PCR analysis and normalized to GAPDH expression. Fold change was calculated using 2^−ΔΔCT^. **(E)** Representative western blotting images of NFATc1 and CTSK after stimulation with or without RANKL and BBM for 5 days. **(F,G)** Quantitative analysis of NFATc1 **(F)** and CTSK **(G)** protein expression normalized to β-actin expression. **(H)** Representative confocal images of the NFATc1 nuclear translocation assay in BMMs treated with or without 4 μM BBM. **(I,J)** Quantitative analysis of the mean NFATc1 fluorescence intensity in the nucleus **(I)** and percentage of NFATc1-positive nuclei **(J)**. **(K)** Representative western blotting images of NFATc1, CTSK, c-Fos and MMP9 after stimulation with or without 4 μM BBM for the indicated time. **(L–O)** Quantitative analysis of the expression levels of NFATc1 **(L)**, CTSK **(M)**, c-Fos **(N)** and MMP9 **(O)** normalized to β-actin expression. *p < 0.05 and **p < 0.01 compared to the control group.

The expression of osteoclast-specific genes, including NFATc1, CTSK, TRAP and MMP9, was examined by quantitative real-time PCR and western blot analysis. The mRNA expression of osteoclast-related genes was inhibited in a dose-dependent manner by BBM (Figures 3A–D), which was confirmed by western blot analysis of NFATc1 and CTSK expression (Fig. E–G). Additionally, RANKL-mediated NFATc1 nuclear translocation was quantitatively analyzed by immunofluorescence staining. The fluorescence intensity of NFATc1 in the nucleus and NFATc1 nuclear translocation were significantly inhibited by 4 μM BBM (Figures 3H–J), which was confirmed by quantifying NFATc1 in the nucleus using western blot analysis (Supplementary Figure S2). We further examined the expression of osteoclast-specific genes at different stages of osteoclast differentiation. Western blot analysis showed that 4 μM BBM downregulated the protein expression levels of NFATc1, CTSK, c-Fos and MMP9 on Days 1, 3 and 5 (Figures 3K–O).

### BBM inhibits RANKL-Induced activation of the NF-κB signaling pathway

**FIGURE 4:**
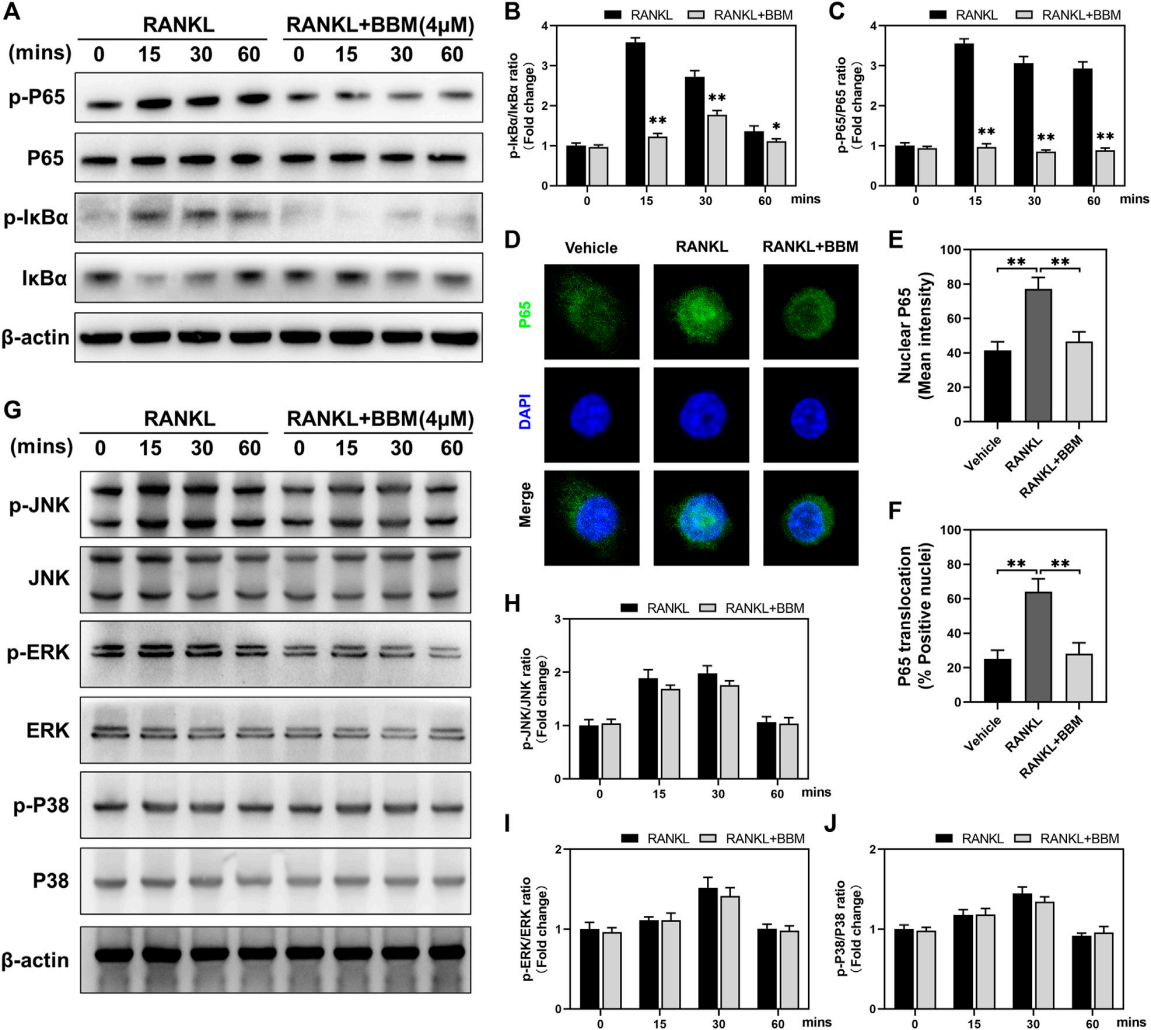
BBM inhibits RANKL-induced activation of the NF-κB signaling pathway. **(A)** Representative western blotting images of P65, p-P65, IκBα and p-IκBα after stimulation with or without 4 μM BBM for the indicated time. **(B–C)** Quantitative analysis of the p-P65/P65 ratio **(B)** and p-IκBα/IκBα ratio **(C)** at each indicated time point. **(D)** Representative confocal images of the P65 nuclear translocation assay in BMMs treated with or without 4 μM BBM. **(E–F)** Quantitative analysis of the mean P65 fluorescence intensity in the nucleus **(E)** and the percentage of P65-positive nuclei **(F)**. **(G)** Representative western blotting images of JNK, p-JNK, ERK, p-ERK, P38 and p-P38 after stimulation with or without 4 μM BBM for the indicated time. **(H–J)** Quantitative analysis of the p-JNK/JNK ratio **(H)**, p-ERK/ERK ratio **(I)** and p-P38/P38 ratio **(J)** at each indicated time point. *p < 0.05 and **p < 0.01 compared to the control group.

To explore the possible mechanisms mediating the inhibitory effect of BBM on osteoclast differentiation, we investigated the early changes in the NF-κB and MAPK pathways, two primary RANKL-induced signaling pathways, during osteoclastogenesis. The protein expression levels of p-P65/P65 and p-IκBα/IκBα at 0, 15, 30, and 60 min after 50 ng/ml RANKL stimulation were significantly suppressed by 4 μM BBM (Figures 4A–C). We further explored RANKL-mediated P65 nuclear translocation by immunofluorescence staining, and the results showed that the fluorescence intensity of P65 in the nucleus and P65 nuclear translocation were significantly inhibited by 4 μM BBM (Figures 4D–F). In addition, the protein expression levels of MAPK pathway components were examined by western blot analysis. No significant difference was found between the control group and BBM treatment group at any timepoint (Figures 4G–J). These results indicated that BBM suppresses RANKL-induced osteoclast differentiation mainly by inhibiting the activation of the NF-κB pathway.

### BBM suppresses bone loss in ovariectomized mice

**FIGURE 5:**
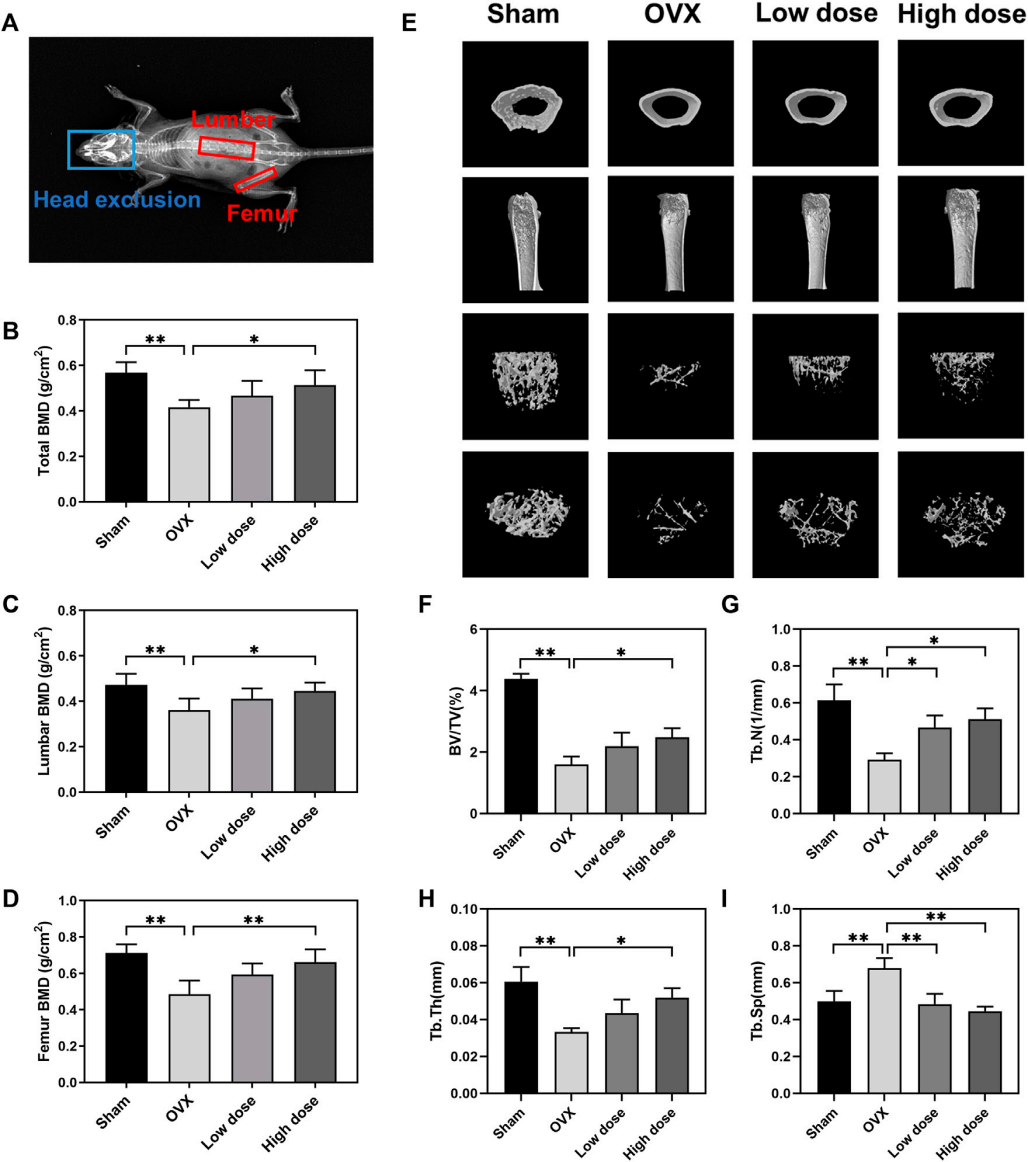
BBM suppresses bone loss in ovariectomized mice. **(A)** Schematic of the BMD analysis using DXA. **(B–D)** Quantitative analysis of the total BMD **(B)**, lumbar BMD **(C)** and femur BMD **(D)** of mice. **(E)** Representative μCT 3D-reconstructed images of distal femurs. **(F–I)** Quantitative analysis of the trabecular bone in the distal femur metaphysis, including BV/TV **(F)**, Tb.N **(G)**, Tb.Th **(H)** and Tb. Sp (i). *p < 0.05 and **p < 0.01 compared to the OVX group.

To assess the therapeutic effect of BBM on osteoporosis, an OVX mouse model was established in C57BL/6J mice followed by treatment with an intraperitoneal injection of BBM every 2 days for 8 weeks. The DXA results showed that the OVX-induced decrease in BMD of the lumbar spine (sham: 0.47 ± 0.05 g/cm^2^; OVX: 0.36 ± 0.05 g/cm^2^; low dose: 0.41 ± 0.05 g/cm^2^; high dose: 0.45 ± 0.04 g/cm^2^), femur (sham: 0.71 ± 0.05 g/cm^2^; OVX: 0.49 ± 0.07 g/cm^2^; low dose: 0.59 ± 0.06 g/cm^2^; high dose: 0.66 ± 0.07 g/cm^2^) and total body (sham: 0.57 ± 0.05 g/cm^2^; OVX: 0.42 ± 0.03 g/cm^2^; low dose: 0.47 ± 0.07 g/cm^2^; high dose: 0.51 ± 0.06 g/cm^2^) was reversed by treatment with 50 mg/kg BBM (Figures 5A–D).

The femurs were harvested for subsequent µCT analysis of trabecular bone in the distal femur metaphysis. Representative μCT 3D reconstruction images of distal femurs showed that BBM effectively suppressed OVX-induced bone loss (Figure 5E). Quantitative analysis demonstrated that BV/TV, Tb.N and Tb.Th were reduced in OVX mice, while Tb. Sp was increased in OVX mice. Treatment with 50 mg/kg BBM significantly rescued the parameters mentioned above, and 25 mg/kg BBM treatment prevented the decrease in Tb.N and the increase in Tb. Sp compared to the OVX group (Figures 5F–I).

### BBM inhibits osteoclastogenesis in ovariectomized mice

**FIGURE 6:**
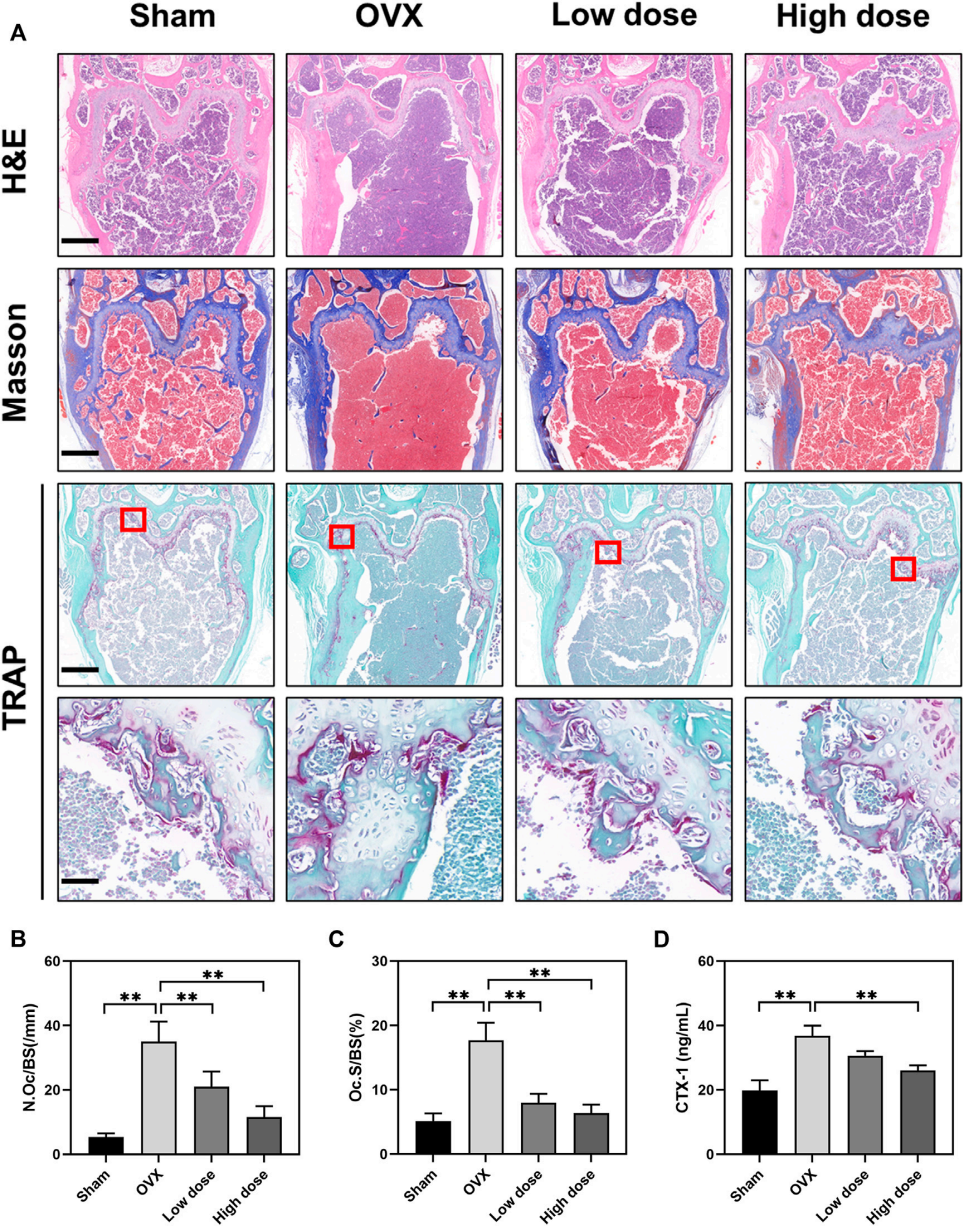
BBM inhibits osteoclastogenesis in ovariectomized mice. **(A)** Representative images of distal femur sections stained with H&E, Masson’s trichrome and TRAP (scale bar = 500 μm; scale bar = 50 μm in the enlarged pictures). **(B–C)** Quantitative analysis of N. Oc/BS **(B)** and Oc S/BS **(C)** in the TRAP staining images of distal femurs. **(D)** Serum levels of the CTX-1 bone resorption marker quantified by ELISA. *p < 0.05 and **p < 0.01 compared to the OVX group.

To further assess the effect of BBM on osteoclast formation *in vivo*, femurs were prepared for histopathological assessment and subjected to H&E, Masson’s trichrome and TRAP staining. The suppressive effect of BBM on OVX-induced bone loss was further confirmed by H&E and Masson’s trichrome staining of the distal femur (Figure 6A). Quantitative analysis of TRAP staining revealed that BBM effectively inhibited osteoclast formation *in vivo* (Figures 6A–C). Furthermore, serum levels of the osteoclast bone resorption marker, CTX-1, were reduced by 50 mg/kg BBM treatment (Figure 6D). These findings indicated that BBM suppresses OVX-induced bone loss by inhibiting osteoclastogenesis and osteoclast bone resorption *in vivo*.

### BBM does not influence osteogenic differentiation *in vitro* or *in vivo*

**FIGURE 7:**
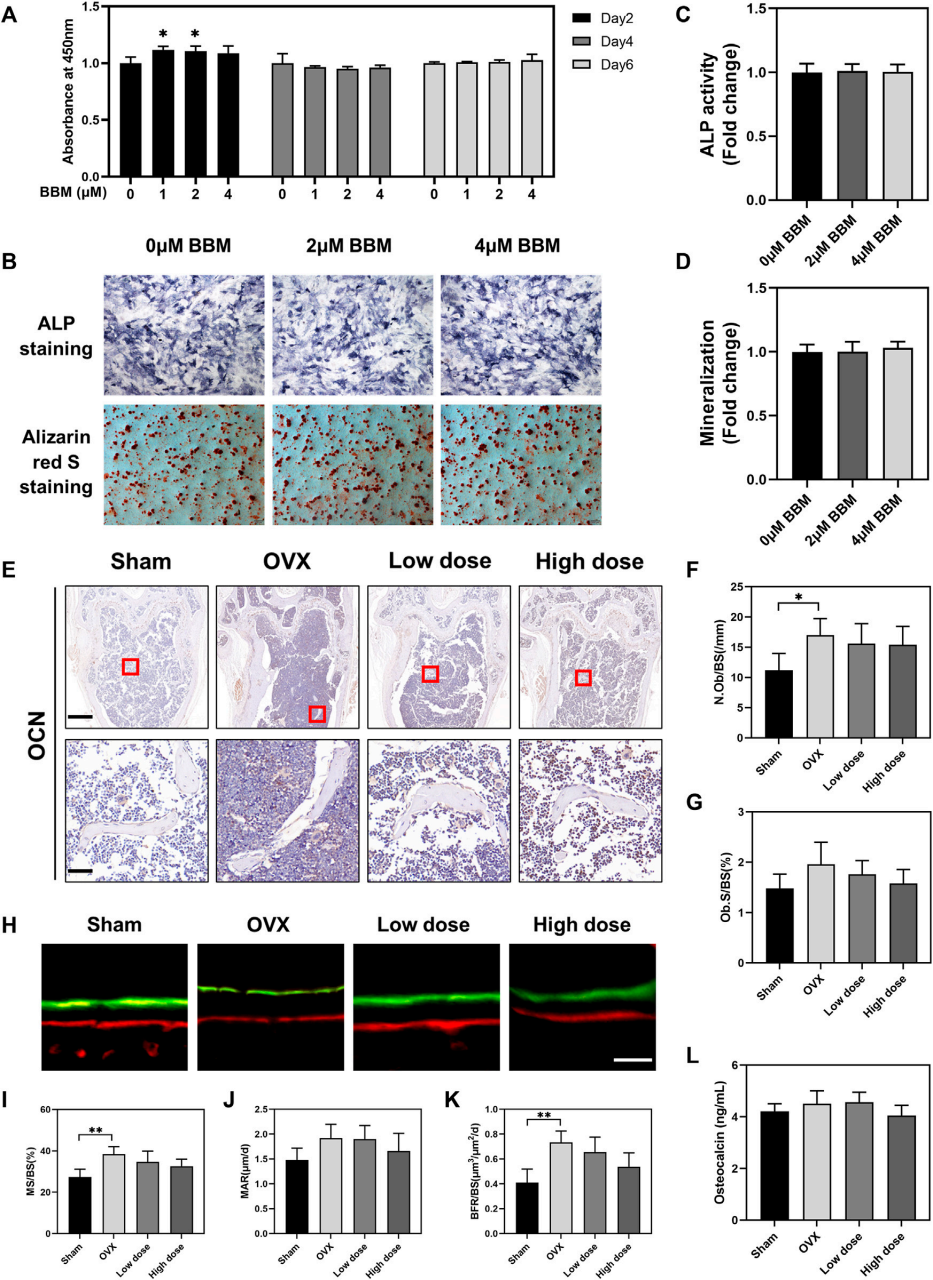
BBM does not influence osteogenic differentiation *in vitro* or *in vivo*. **(A)** MC3T3-E1 cells were treated with BBM (0, 1, 2 and 4 μM) for 2, 4 and 6 days. Cell viability was determined by CCK-8 assay. **(B)** Representative images of ALP and ARS staining in MC3T3-E1 cells (scale bar = 500 μm). **(C)** Quantitative analysis of ALP activity in the ALP staining images. **(D)** Quantitative analysis of mineralization in the ARS staining images. **(E)** Representative images of distal femur sections immunohistochemically stained for OCN (scale bar = 500 μm; scale bar = 50 μm in the enlarged pictures). **(F,G)** Quantitative analysis of N. Ob/BS **(F)** and Ob. S/BS **(G)** in the OCN staining images of distal femurs. **(H)** Representative fluorescence images of calcein–Alizarin red S labeling (scale bar = 20 μm). **(I–K)** Quantitative analysis of MS/BS **(I)**, MAR **(J)** and BFR/BS **(K)** in the double-labeling images. **(L)** Serum levels of the OCN bone formation marker quantified by ELISA. *p < 0.05 and **p < 0.01 compared to the control group.

In addition to osteoclasts and bone resorption, the effect of BBM on osteogenic differentiation was also assessed *in vitro* and *in vivo*. A CCK-8 assay was performed *in vitro* to assess the toxic effects of BBM on MC3T3-E1 cells, and no obvious cytotoxicity was observed after 6 days of incubation with 1, 2, and 4  μM BBM (Figure 7A). Furthermore, the osteogenesis and mineralization of MC3T3-E1 cells under the control and BBM treatments were assessed by ALP and ARS staining, and no significant difference was observed (Figures 7B–D). We examined the effect of BBM on osteogenesis *in vivo* by IHC analysis of the OCN bone formation marker (Figures 7E–G), calcein–Alizarin red S labeling analysis (Figures 7H–K) and serum OCN levels (Figure 7L). The results revealed no significant difference between the OVX and BBM (25 mg/kg or 50 mg/kg) treatment groups. Taken together, these findings demonstrated that BBM effectively suppresses osteoclast differentiation and OVX-induced bone loss without affecting osteogenic differentiation.

### *In vivo* toxicity evaluation

**FIGURE 8:**
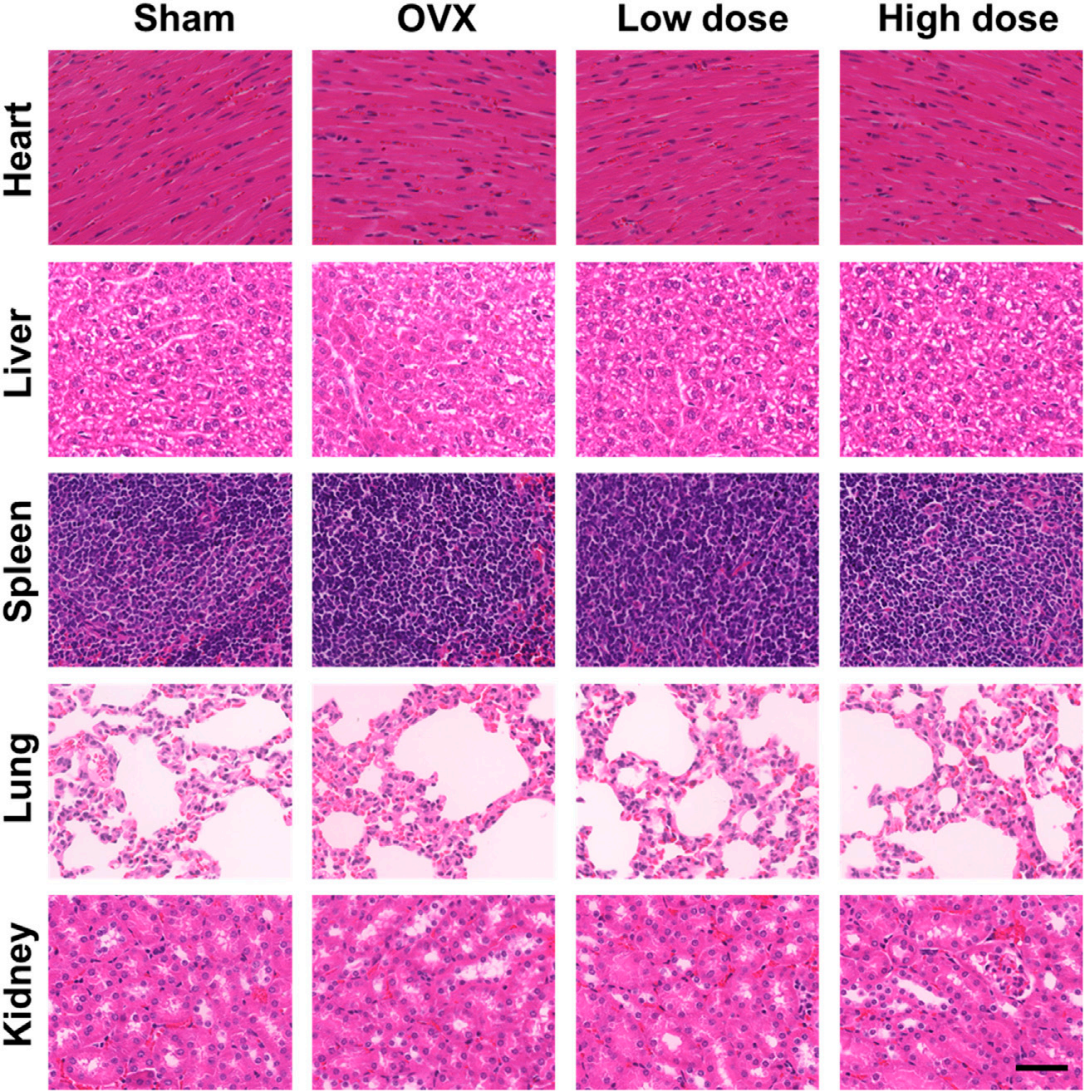
*In vivo* toxicity evaluation. No histologic evidence of organ toxicity was detected at the applied doses in the H&E staining of the heart, liver, spleen, lung and kidney tissues of mice from each group (scale bar = 50 μm).

To assess organ toxicity under BBM treatment, the heart, liver, spleen, lung and kidney tissues of mice were stained with H&E. No obvious pathological changes in organ tissues were observed (Figure 8), which indicated that BBM had no significant organ toxicity at the applied doses.

## Discussion

Bone is a dynamic tissue undergoing a continuous remodeling process, which is maintained by a balance between osteoclast-mediated bone resorption and osteoblast-mediated bone formation (Hadjidakis and Androulakis, 2006; Amend et al., 2015). With aging, bone homeostasis is gradually disrupted due to excessive bone resorption by osteoclasts relative to bone formation by osteoblasts, resulting in osteoporosis and increased susceptibility to fracture (Demontiero et al., 2012; Farr and Khosla, 2015). For patients with osteoporosis, pharmacological treatments are necessary to increase bone mass and reduce fracture incidence. Current drugs approved to treat osteoporosis broadly fall into two categories, namely, antiresorptive drugs and anabolic drugs. Anti-resorptive drugs are the most commonly used (Li et al., 2021), mainly including bisphosphonates (first-line drugs to treat osteoporosis by inducing the apoptosis of osteoclasts), denosumab (selectively inhibits RANKL binding to RANK) and estrogen (estrogen replacement therapy) (Drake et al., 2008; Levin et al., 2018; Kendler et al., 2022). However, the prolonged use of these drugs is limited by decreased efficacy and severe side effects. Natural compounds extracted from plants have been considered an alternative treatment option for osteoporosis as they inhibit the abnormal behavior of osteoclasts without apparent side effects and overcome the limitations of the currently used treatments (Wang et al., 2017; Suvarna et al., 2018). In the present study, we explored the effect of BBM, a natural product isolated from traditional Chinese medicine, on osteoclast differentiation, fusion and bone resorption activity *in vitro* as well as OVX-induced osteoporosis *in vivo*. BBM attenuated osteoclast activity by inhibiting the activation of the NF-κB signaling pathway *in vitro* and prevented OVX-induced bone loss *in vivo*.

The NF-κB and MAPK signaling pathways are crucial for RANKL-induced osteoclastogenesis. Upon binding of RANKL and RANK, intracellular NF-κB and MAPK signaling pathways are activated and subsequently drive osteoclast differentiation by regulating downstream signal transduction. Previous studies have suggested that inhibition of the NF-κB and MAPK signaling pathways significantly decreases osteoclast formation and bone resorption activity (Park et al., 2017; Li et al., 2020; Yang et al., 2021), which was consistent with our results. In the present study, western blot analysis and osteoclast differentiation assays showed that BBM suppressed osteoclastogenesis and osteoclast resorption by inhibiting the NF-κB signaling pathway. Immunofluorescence staining of P65 confirmed the inhibitory effect of BBM on P65 nuclear translocation. Nevertheless, the present study demonstrated that BBM did not affect the MAPK signaling pathway. Jia *et al.* reported that BBM exerts anti-inflammatory effects by inhibiting the NF-κB and MAKP signaling pathways (Jia et al., 2017). Similarly, Liu *et al.* showed that BBM inhibits the inflammatory response and reduces liver injury *via* inactivation of the NF-κB, STAT3 and MAPK/ERK pathways (Liu et al., 2020). The effective concentration of BBM applied in their *in vitro* studies was 10 μM, while the maximum concentration applied in the present study was 4 μM. Therefore, the concentration of BBM, together with different cell types and treatment conditions, may account for this difference in the MAPK pathway.

NFATc1, a downstream nuclear transcription factor induced by the activated NF-κB pathway, plays an essential role in regulating the expression of osteoclast-related genes during RANKL-induced osteoclastogenesis, such as TRAP, CTSK and MMP9, which are involved in the regulation of osteoclast differentiation, fusion and activation (Asagiri and Takayanagi, 2007; Park et al., 2017). Additionally, an activator protein (AP)-1 complex containing c-Fos is necessary to enable the robust induction of NFATc1 by cooperating with NFATc1 and thereby activating several target genes involved in osteoclast formation and resorptive function (Asagiri and Takayanagi, 2007). The present study revealed that BBM inhibited the protein expression of NFATc1 and c-Fos as well as the nuclear translocation of NFATc1 in RANKL-treated BMMs. Furthermore, BBM suppressed the expression of downstream transcriptional targets during osteoclastogenesis, including CTSK and MMP9, which degrade the bone matrix and contribute to osteoclast bone resorption. These results further demonstrated that BBM may suppress osteoclast formation and bone resorption by downregulating the expression of NFATc1 and its downstream genes.

In the present study, we established an OVX mouse model to assess whether BBM prevents OVX-induced bone loss *in vivo*. The results of DXA and µCT analysis showed that BBM prevented OVX-induced bone loss, which was further confirmed by H&E and Masson’s trichrome staining. TRAP staining demonstrated that BBM treatment significantly reduced osteoclast number and surface area, indicating a protective role of BBM against osteoporosis by inhibiting osteoclast formation and bone resorption function.

Currently, antiresorptive drugs are the most common treatment for osteoporosis (Reid and Billington, 2022). However, the decrease in osteoclasts may lead to a decrease in osteogenesis, thus limiting the therapeutic efficacy of antiresorptive drugs. For example, bisphosphonate drugs are used clinically as antiresorptive drugs that inhibit osteoclastic bone resorption by disrupting intracellular signaling and inducing apoptosis of osteoclasts (Rogers et al., 2011). Recent studies have reported that bisphosphonates reduce bone formation by inhibiting surface-mediated osteogenesis and impairing the onset of bone formation after resorption (Lotz et al., 2020; Jensen et al., 2021). Therefore, we also investigated the effect of BBM on osteoblast differentiation and bone formation, and we did not observe a significant difference between the BBM treatment group and the control group, suggesting that BBM may effectively suppress osteoclast differentiation and bone resorption without affecting osteogenesis.

In summary, the present study demonstrated that BBM has a protective role against osteoporosis by inhibiting osteoclastogenesis and bone resorption function without affecting osteogenesis or causing systemic toxicity. The present study had several limitations. First, we did not investigate the effect of BBM at a higher concentration on osteogenesis or organ toxicity. Second, there was a lack of positive controls to better evaluate the therapeutic effect, such as bisphosphonates, when investigating the inhibitory effect of BBM on osteoclastogenesis *in vitro*. In addition, the calcium signaling pathway also plays a crucial role in osteoclastogenesis, and further studies are required to investigate whether BBM affects the Ca^2+^-NFATc1 pathway.

## Conclusion

In conclusion, the present study demonstrated that BBM inhibits osteoclastogenesis and bone resorption function *in vitro* as well as prevents OVX-induced bone loss *in vivo*. The inhibitory effect of BBM on osteoclastogenesis is achieved by inhibiting RANKL-induced activation of the NF-κB signaling pathway. In addition, BBM protects against osteoporosis by inhibiting osteoclast formation and bone resorption function without affecting osteogenesis in the OVX mouse model. Thus, these findings suggested that BBM may represent a novel promising treatment strategy for osteoporosis.

## Data Availability

The datasets presented in this study can be found in online repositories. The names of the repository/repositories and accession number(s) can be found in the article/Supplementary Material.
