# Supplementary material for: Berbamine inhibits RANKL- and M-CSF-mediated osteoclastogenesis and alleviates ovariectomy-induced bone loss
Source: Front Pharmacol. 2022 Nov 2;13:1032866. doi: 10.3389/fphar.2022.1032866 (PMC9666778; doi:10.3389/fphar.2022.1032866)
Supplement: Supplementary file 1 [file DataSheet1.docx]

Supplementary Material

**
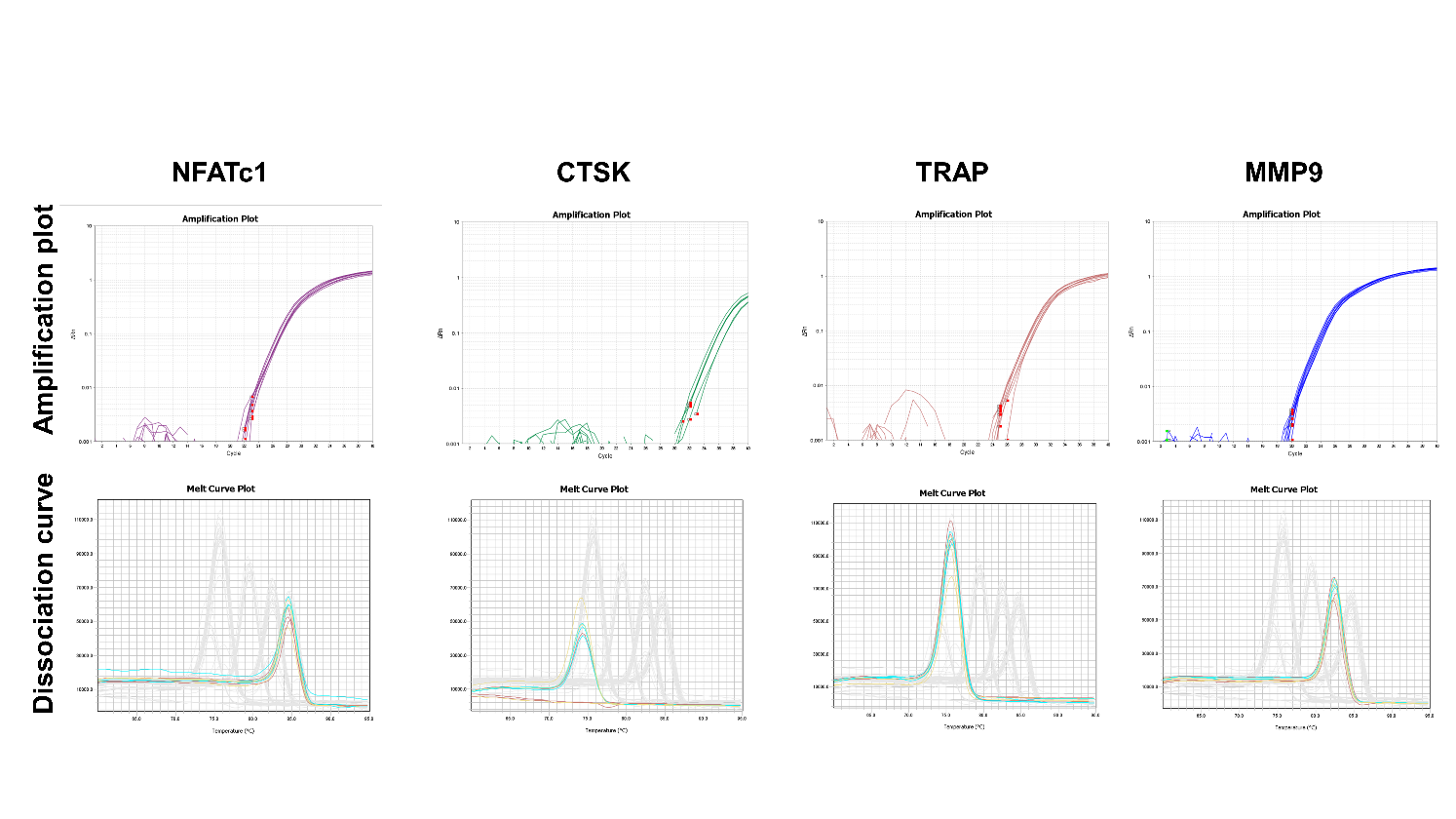
**

**Supplementary Figure 1.** The amplification plot and dissociation curve of the gene expression levels of NFATc1, CTSK, TRAP and MMP9 determined by quantitative real-time PCR analysis.


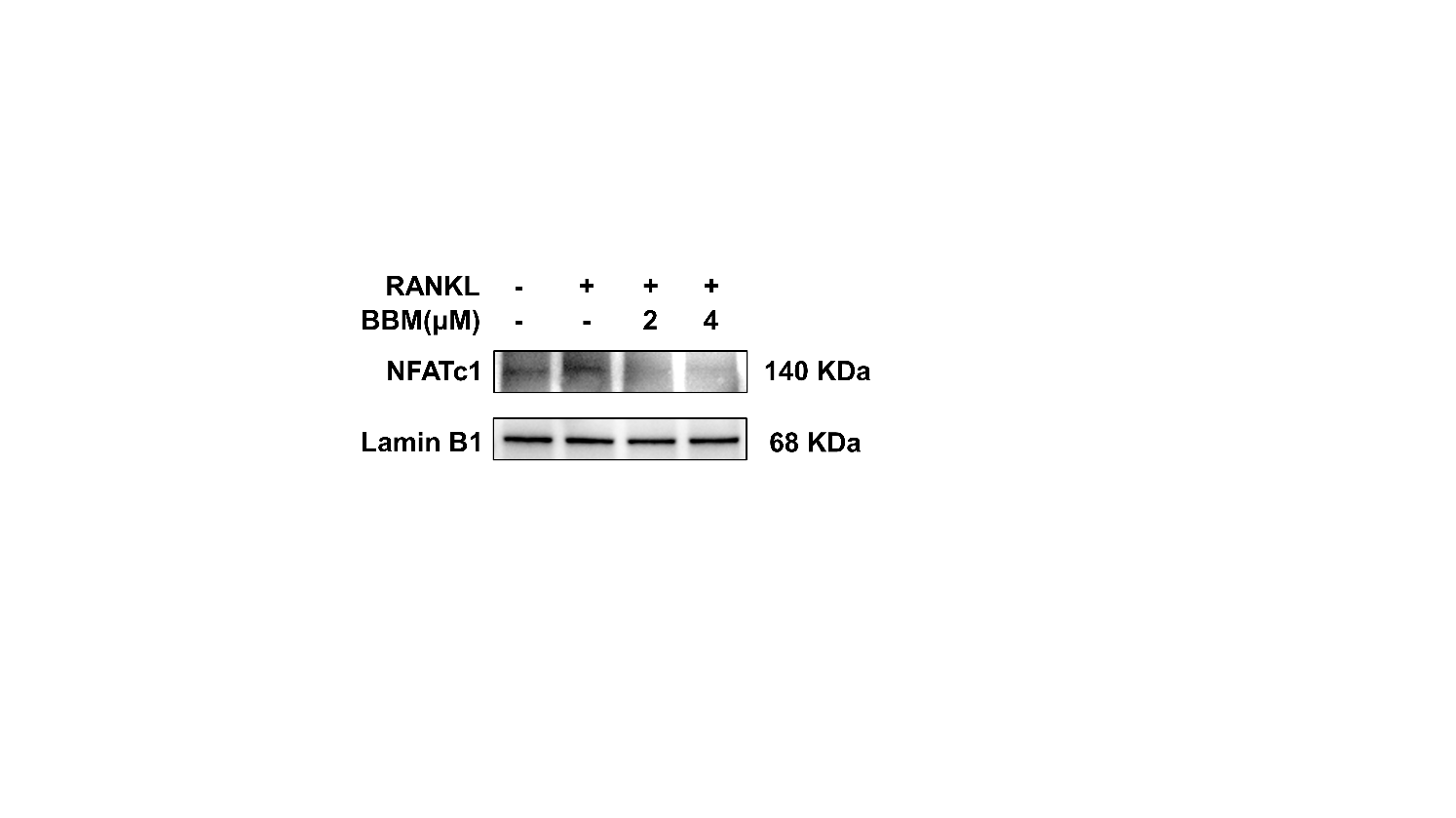


**Supplementary Figure 2.** Quantification of NFATc1 in the nucleus using western blot analysis.
